# Announcements

**Published:** 2015-04-03

## STD Awareness Month—April 2015

April is STD Awareness Month, an annual observance that focuses on ways to prevent some of the nearly 20 million new cases of STDs occurring in the United States each year (*1*). CDC’s STD prevention program emphasizes the most effective tools to protect one’s health and prevent the spread of all STDs, including HIV: 1) learn the facts about STDs; 2) make lifestyle changes that reduce risk; 3) get regular STD testing, as needed, and 4) seek prompt treatment.

STDs affect persons of all ages, but particularly the young. CDC estimates that half of all new infections are among people aged 15–24 (*1*). STD tests aren’t always part of a regular doctor’s visit, and many doctors may not offer young patients an HIV or STD test unless the patient asks for one. Patients who get tested for STDs and are aware of their STD status can better protect their own health and the health of their sexual partner(s). If not treated, some STDs can lead to serious health problems. Learning resources for clinicians, patients, and community members about STDs are available from CDC at http://www.cdc.gov/std/sam.

## References

[1] Satterwhite CL, Torrone E, Meites E (2013). Sexually transmitted infections among US women and men: prevalence and incidence estimates, 2008. Sex Transm Dis.

